# Single Incision Laparoscopic Cholecystectomy by Using a 2 mm Atraumatic Grasper without Trocar

**DOI:** 10.1155/2011/761315

**Published:** 2011-09-11

**Authors:** Kamil Gulpinar, Suleyman Ozdemir, S. Erpulat Ozis, Turgut Aydin, Atila Korkmaz

**Affiliations:** Department of Surgery, Ufuk University, 06520 Ankara, Turkey

## Abstract

*Purpose*. We present our experience in single incision laparoscopic cholecystectomy by using a grasper directly without using a trocar in five patients. *Methods and Results*. The technique involves the use of Karl Storz 27290F grasper in order to perform gallbladder retraction in single port cholecystectomy. The grasper was introduced directly into the skin through abdominal wall without using any trocar and used to mobilize gallbladder whenever needed during surgery without causing any perforation or leakage of the gallbladder. There were no intraoperative and postoperative complications in 5 patients with the advantages of shorter operation time and almost invisible postoperative skin scar formation. *Conclusion*. We claim that the use of this instrument in SILS surgery might be advantageous than the conventional placement of sutures for the gallbladder mobilization.

## 1. Introduction

Single incision laparoscopic surgery (SILS) for cholecystectomy procedure has been introduced as early as 1999 [1] to achieve less pain, less scarring, and less hospitalization period. One of the major difficulties of this procedure seems to be the traction of the gallbladder in order to expose the tissues during operation without additional ports. The use of transabdominal 2–0 nylon sutures attached to Keith needles [2] and the use of a Kirschner wire hook introduced through subcostal area [3] are reported methods used for traction of gallbladder and better exposition of the Calot triangle. However, all these techniques consist puncture of the gallbladder with sharp needles especially if it is distended and carry the risk of bile leakage and contamination afterwards. They also have limited ability to mobilization whenever needed.

Herein, we describe our technique in establishing single port access for cholecystectomy in five patients that involves the use of a 2 mm in diameter grasper, Karl Storz 27290F, that is generally used by urologists for percutaneous nephrolithiasis intervention.

## 2. Material and Methods

**Figure 1:**
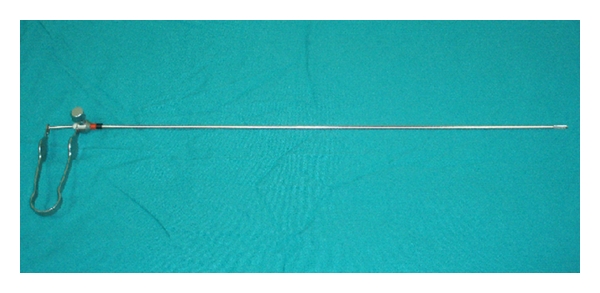
A minigrasper, Karl Storz 27290F, 2 mm in diameter.

**Figure 2:**
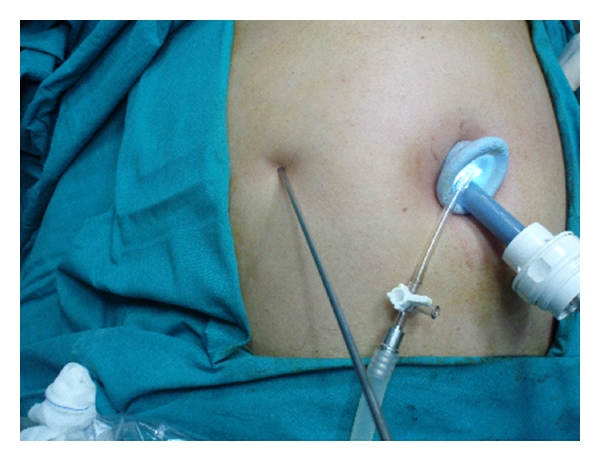
Karl Storz 27290F, 2 mm in diameter, was inserted thoroughly an incision, 1 to 2 mm in length, created at the transaction point of the midclavicular vertical line and umbilical horizontal line.

**Figure 3:**
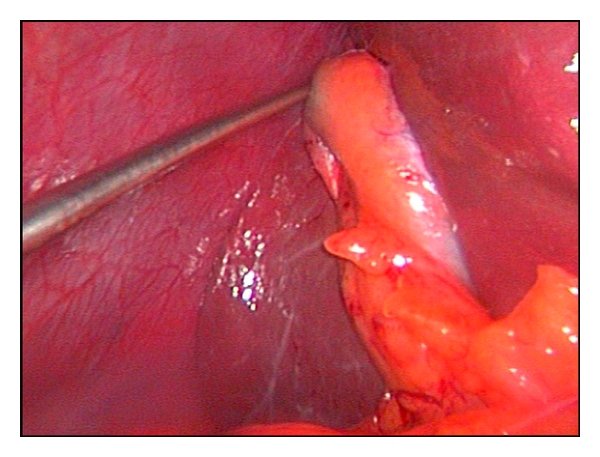
Karl Storz 27290F, 2 mm in diameter, grasper is used to hold the fundus of the gallbladder gently and push the organ in a cephalad direction to visualize Calot's triangle.

After induction of general anesthesia, an umbilical skin incision, 1.5 cm in length, is made vertically and a Covidien SILS port having 5 mm–12 mm four holes is inserted to the peritoneal cavity under direct vision and pneumoperitoneum is created. A 10 mm 30° videoscope is placed through the port. Thereafter, a minigrasper, Karl Storz 27290F, 2 mm in diameter (Figure 1), was inserted thoroughly an incision, 2 mm in length, created at the transaction point of the midclavicular vertical line and umbilical horizontal line (Figure 2). It is used to hold the fundus of the gallbladder gently and push the organ in an cephalad direction to visualize Calot's triangle (Figure 3). This atraumatic device helped to mobilize gallbladder whenever needed during surgery without causing any wound or leakage. Then, the dissection of the gallbladder was completed in a standard fashion from bottom to top [4] and after the organ was dissected free from the liver, it was removed directly through the umbilical incision. 

No complications including gas leakage around the minigrasper were encountered during and after the surgery. Patients were discharged from the hospital within 24 hours. Followup was done in the first month and minimal scarring was observed in the umbilicus and the minigraspers' small incision was almost invisible.

## 3. Discussion

Single Incision Laparoscopic Cholecystectomy (SILC) was first described by Navarra et al. [4] in 1997, followed by Piskun and Rajpal [1] in 1999. Since then, SILC technique has been performed by many clinicians with promising results and many advantages like few complications, acceptable operation period, less scarring, less pain, and quicker recovery [2].

There are many technical challenges in SILC surgery as one of them seems to be the retraction of the fundus cephalad and traction of the Hartmann's pouch laterally to ensure safe cholecystectomy. For this purpose, some surgeons use transabdominal 2–0 nylon sutures attached to Keith needles for fixation and assist gallbladder retraction [2] and some other used Kirschner wire hook which is introduced through subcostal area to pull gallbladder in an upright direction during the operation [3]. Yet, all these techniques consist puncture of the gallbladder with sharp needles especially if it is distended and carry the risk of bile leakage and contamination afterwards. They also have limited ability to mobilize whenever needed. To achieve gallbladder retraction and cephalad traction, we have used Karl Storz 27290F, instead of sutures or Kirschner wire hook. This minigrasper is used not only in performing sufficient traction of the gallbladder in an upright position, but also it can allow good mobilization of the gallbladder in order to dissect Calot's triangle safely. Use of this instrument has led no perforation and leakage of bile in the patients. Through a very small incision, 2 mm in length, without using any port, it has been introduced directly into the abdomen under camera control and made the dissection trouble-free as the manipulation of the gallbladder could be done easily to every direction needed. No gas leakage has been noted around the instrument after direct introduction into the abdomen in all cases. The most remarking effect of using this technique was its influence on the operation time. Previous reports using port systems or transabdominal sutures declare their mean operating time to be approximately at least 30 min [2] to 70 min [3]. Our procedure even on the first patient took only 40 min to conclude without any complications. We believe learning curve would be low and it would be possible to significantly decline operation periods in following patients.

In conclusion, with the help of this instrument the operation period may be lessened, less scarring is achieved and surgical procedure is done more easily and safely without any additional costs. We propose to use this minigrasper in performing SILC operation safely.
